# Supplementary material for: Late Pleistocene Expansion of Small Murid Rodents across the Palearctic in Relation to the Past Environmental Changes
Source: Genes (Basel). 2021 Apr 26;12(5):642. doi: 10.3390/genes12050642 (PMC8145813; doi:10.3390/genes12050642)
Supplement: Supplementary file 1 [file genes-12-00642-s001.zip › File S2.pdf]

Late Pleistocene Expansion of Small Murid Rodents across the Palearctic in Relation to the Past Environmental Changes

Kozyra K., Zając T. M., Ansorge H., Wierzbicki H., Moska M., Stanko M., Stopka P.

Supplementary Materials S2

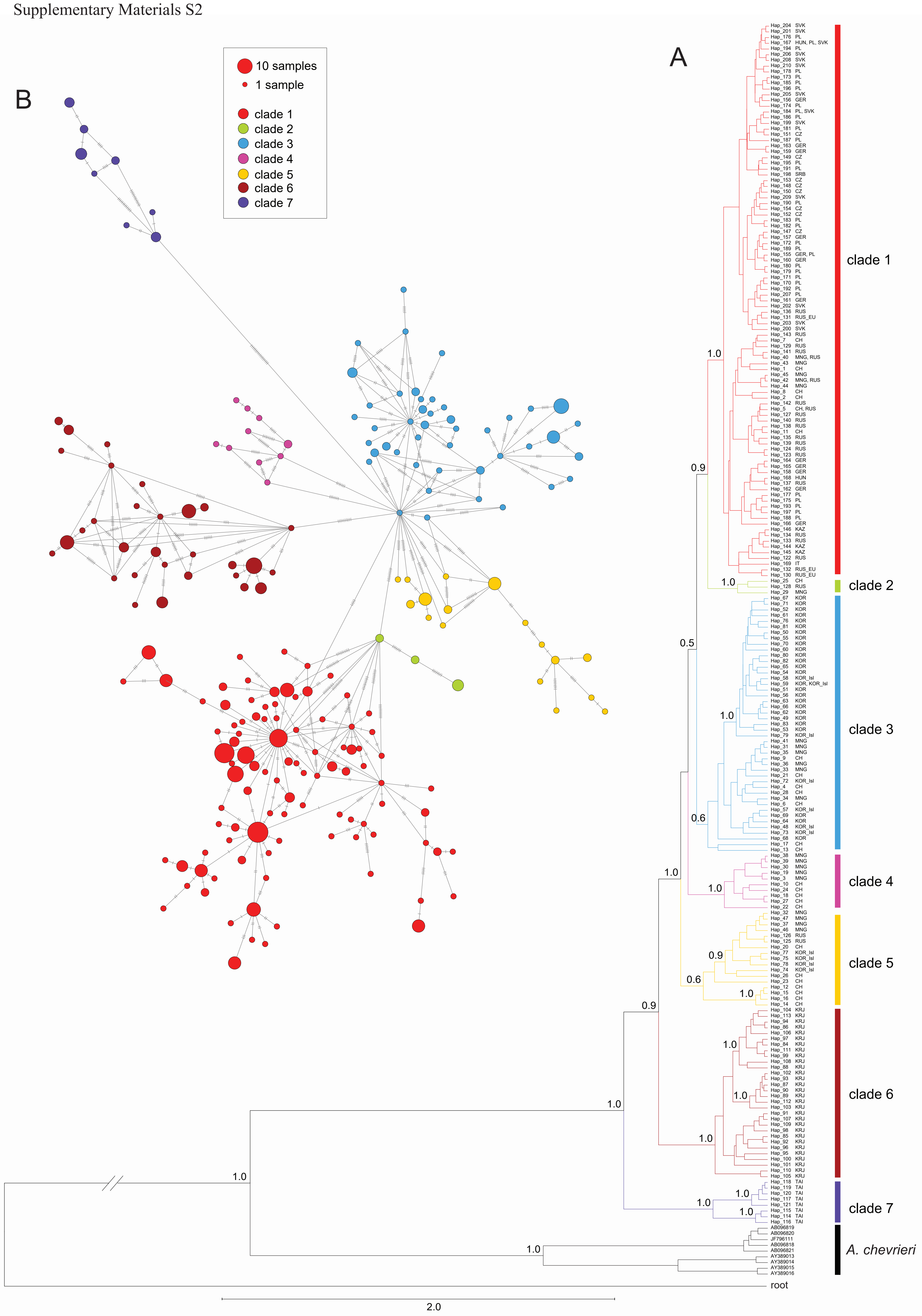

Figure S1. The phylogenetic tree of striped-field mouse *A. agrarius* based on the Bayesian Inference (BI) in BEAST (A) and Minimum Spanning Network generated in PopArt 1.7 (B).
