# Supplementary material for: Late Pleistocene Expansion of Small Murid Rodents across the Palearctic in Relation to the Past Environmental Changes
Source: Genes (Basel). 2021 Apr 26;12(5):642. doi: 10.3390/genes12050642 (PMC8145813; doi:10.3390/genes12050642)
Supplement: Supplementary file 1 [file genes-12-00642-s001.zip › File S3.pdf]

Late Pleistocene Expansion of Small Murid Rodents across the Palearctic in Relation to the Past Environmental Changes

Kozyra K, Zając T.M., Ansorge H., Wierzbicki H., Moska M., Stanko M., Stopka P.

Supplementary materials S3

Demographic and evolutionary scenarios within the three models using the ABC method implemented in DIYABC.

Table S4. Demographic models and scenarios with parameters used in the ABC analysis in DIYABC.

Parameters	Model 1		Model 2	
t_1			Uniform (1000-8800)	
t_2	Uniform (4500-30000)	$t_2>t_1$	Uniform (4500-30000)	$t_2>t_1$
t_3	Uniform (22000-60000)	$t_3>t_2$	Uniform (22000-60000)	$t_3>t_2$
t_4	Uniform (44000-120000)	$t_4>t_3$	Uniform (44000-120000)	$t_4>t_3$
Scenario 1	Constant population		Constant population	
N1	Uniform (10000-15000000)		Uniform (10000-15000000)	
Scenario 2	Holocene expansion		Late Holocene expansion	
N_2	Uniform (10000- 15000000)		Uniform (10000- 15000000)	
N_{2a}			Uniform (1000-10000000)	$N_{2a}<N_2$
N_{2b}	Uniform (10000-5000000)	$N_{2b}<N_2$		
Scenario 3	Holocene decline		Early/Middle Holocene bootleneck	
N_3	Uniform (10000- 15000000)		Uniform (10000- 15000000)	
N_{3a}			Uniform (1000-5000000)	$N_{3a}<N_3$
N_{3b}	Uniform (10000-20000000)	$N_{3b}>N_3$	Uniform (10000- 15000000)	$N_{3b}>N_{3a}$
Scenario 4	Holocene decline after glacial expansion		Middle/Early Holocene bottleneck after Middle/Late glacial expansion	
N_4	Uniform (10000-15000000)		Uniform (10000- 15000000)	
N_{4a}			Uniform (1000-5000000)	$N_{4a}<N_4$
N_{4b}	Uniform (10000-15000000)	$N_{4b}>N_4$	Uniform (10000- 15000000)	$N_{4b}>N_{4a}$
N_{4d}	Uniform (1000- 1000000)	$N_{4d}<N_{4b}$	Uniform (1000-5000000)	$N_{4d}<N_{4b}$
Scenario 5	Holocene expansion after glacial decline		Late Holocene expansion preceded by late glacial and postglacial bootleneck	
N_5	Uniform (10000- 15000000)		Uniform (10000- 15000000)	
N_{5a}			Uniform (1000-5000000)	$N_{5a}<N_5$
N_{5b}	Uniform (1000- 5000000)	$N_{5b}<N_5$		
N_{5c}			Uniform (10000- 15000000)	$N_{5c}>N_{5a}$
N_{5d}	Uniform (10000- 15000000)	$N_{5d}>N_{5b}$		
Scenario 6	Bootleneck during LGM			

N_6	Uniform (50000- 15000000)		
N_{6b}	Uniform (1000- 1000000)	$N_{6b}<N_6$	
N_{6c}	Uniform (50000- 15000000)	$N_{6c}>N_{6b}$	

Table S5. Divergence model (Model 3) parametrization of East Asiatic populations, used in the ABC analysis in DIYABC.

Parameters	Model 3		
t_1	Uniform (10000-500000)		
t_2	Uniform (20000-500000)	$t_2 \geq t_1$	
N1, N2, N3	Uniform (20000- 2000000)	N2≥Na2<br>N3≥Na2	N1 - clade C5 (China);<br>N2 - clade C3 and C5 (South Korea - remote islands;<br>N3 - clade C3 - South Korea (Korean Peninsula);
Na1, Na2	Uniform (20000- 2000000)	Na2<Na1	Na1, Na2: ancestral populations
r	Uniform (0.001-0.999)		

Table S6. Comparison of 6 scenarios in 1st model using Logistic approach as probability index (in grey column the most probable scenario)

**Model 1 - Logistic approach results**						
**Clade 1**						
n	Scenario 1	Scenario	Scenario 3	Scenario 4	Scenario 5	Scenario 6
5000	0.0059 [0.0000,0.1355]	0.0011 [0.0000,0.1313]	0.0003 [0.0000,0.1306]	0.4880 [0.2593,0.7166]	0.3326 [0.1406,0.5246]	0.1721 [0.0624,0.2818]
10000	0.0049 [0.0000,0.0183]	0.0015 [0.0000,0.0147]	0.0005 [0.0000,0.0138]	0.7844 [0.6982,0.8706]	0.1438 [0.0748,0.2127]	0.0650 [0.0294,0.1006]
15000	0.0043 [0.0005,0.0081]	0.0014 [0.0000,0.0047]	0.0007 [0.0000,0.0040]	0.8747 [0.8335,0.9160]	0.0822 [0.0494,0.1150]	0.0367 [0.0193,0.0540]
20000	0.0040 [0.0020,0.0061]	0.0013 [0.0000,0.0027]	0.0006 [0.0000,0.0021]	0.9102 [0.8849,0.9355]	0.0568 [0.0370,0.0766]	0.0270 [0.0157,0.0383]
25000	0.0042 [0.0026,0.0058]	0.0013 [0.0006,0.0019]	0.0005 [0.0000,0.0013]	0.9317 [0.9148,0.9486]	0.0415 [0.0285,0.0545]	0.0209 [0.0130,0.0287]
30000	0.0043 [0.0029,0.0057]	0.0012 [0.0008,0.0016]	0.0005 [0.0000,0.0011]	0.9459 [0.9339,0.9579]	0.0320 [0.0228,0.0412]	0.0161 [0.0105,0.0216]
**Clade 3**						
5000	0.0029 [0.0000,0.0621]	0.0001 [0.0000,0.0599]	0.0153 [0.0000,0.0779]	0.7646 [0.4556,1.0000]	0.1630 [0.0000,0.4601]	0.0542 [0.0000,0.1614]
10000	0.0017 [0.0000,0.0105]	0.0001 [0.0000,0.0091]	0.0046 [0.0000,0.0149]	0.8854 [0.7738,0.9969]	0.0785 [0.0000,0.1796]	0.0297 [0.0000,0.0676]
15000	0.0014 [0.0000,0.0059]	0.0002 [0.0000,0.0047]	0.0036 [0.0000,0.0092]	0.9100 [0.8409,0.9792]	0.0649 [0.0010,0.1288]	0.0198 [0.0000,0.0398]
20000	0.0015 [0.0000,0.0047]	0.0003 [0.0000,0.0035]	0.0028 [0.0000,0.0067]	0.9195 [0.8670,0.9720]	0.0610 [0.0116,0.1105]	0.0149 [0.0022,0.0276]
25000	0.0015 [0.0000,0.0040]	0.0003 [0.0000,0.0027]	0.0022 [0.0000,0.0051]	0.9266 [0.8848,0.9685]	0.0570 [0.0172,0.0968]	0.0123 [0.0032,0.0214]
30000	0.0016 [0.0000,0.0035]	0.0003 [0.0000,0.0022]	0.0019 [0.0000,0.0041]	0.9318 [0.8972,0.9665]	0.0534 [0.0203,0.0864]	0.0110 [0.0037,0.0183]

Table S7. Comparison of 5 scenarios in 2nd model using Logistic approach as probability index (in grey column the most probable scenario)

Model 2 - Logistic approach results					
**Clade 1**					
n	Scenario 1	Scenario 2	Scenario 3	Scenario 4	Scenario 5
5000	0.0130 [0.0000,0.0301]	0.0000 [0.0000,0.0133]	0.0000 [0.0000,0.0000]	0.8127 [0.7489,0.8765]	0.1743 [0.1114,0.2372]
10000	0.0147 [0.0057,0.0238]	0.0010 [0.0000,0.0034]	0.0000 [0.0000,0.0000]	0.9062 [0.8815,0.9310]	0.0781 [0.0552,0.1010]
15000	0.0193 [0.0110,0.0275]	0.0019 [0.0000,0.0039]	0.0005 [0.0000,0.0019]	0.9266 [0.9111,0.9420]	0.0517 [0.0392,0.0642]
20000	0.0259 [0.0175,0.0342]	0.0029 [0.0008,0.0050]	0.0005 [0.0000,0.0014]	0.9231 [0.9097,0.9364]	0.0477 [0.0380,0.0574]
25000	0.0328 [0.0243,0.0412]	0.0040 [0.0018,0.0063]	0.0005 [0.0000,0.0012]	0.9158 [0.9033,0.9282]	0.0470 [0.0387,0.0553]
**Clade 3**					
5000	0.2339 [0.0197,0.4481]	0.0832 [0.0000,0.2445]	0.0193 [0.0000,0.1177]	0.5212 [0.2627,0.7796]	0.1424 [0.0000,0.3016]
10000	0.2360 [0.1062,0.3659]	0.1140 [0.0000,0.2394]	0.0138 [0.0000,0.0836]	0.4393 [0.2761,0.6025]	0.1969 [0.0654,0.3284]
15000	0.2107 [0.1215,0.2999]	0.1117 [0.0231,0.2002]	0.0192 [0.0000,0.0682]	0.4566 [0.3345,0.5788]	0.2018 [0.1003,0.3033]
20000	0.2060 [0.1354,0.2766]	0.1052 [0.0380,0.1724]	0.0208 [0.0000,0.0608]	0.4589 [0.3610,0.5569]	0.2090 [0.1243,0.2936]
25000	0.2003 [0.1425,0.2581]	0.1000 [0.0462,0.1539]	0.0217 [0.0000,0.0547]	0.4677 [0.3858,0.5497]	0.2102 [0.1384,0.2820]

Table S8. Comparison of 6 scenarios in 3rd model using Logistic approach as probability index (in grey column the most probable scenario)

Model 3 - Logistic approach						
n	scenario 1	scenario 2	scenario 3	scenario 4	scenario 5	scenario 6
6000	0.2589 [0.1949,0.3228]	0.0854 [0.0307,0.1400]	0.1566 [0.0686,0.2445]	0.3491 [0.2773,0.4210]	0.0646 [0.0079,0.1213]	0.0854 [0.0295,0.1413]
12000	0.2429 [0.2001,0.2857]	0.0831 [0.0470,0.1193]	0.1634 [0.1041,0.2227]	0.3417 [0.2917,0.3917]	0.0643 [0.0269,0.1018]	0.1045 [0.0672,0.1418]
18000	0.2419 [0.2078,0.2760]	0.0886 [0.0605,0.1167]	0.1631 [0.1167,0.2095]	0.3302 [0.2905,0.3699]	0.0632 [0.0340,0.0924]	0.1130 [0.0834,0.1425]
24000	0.2429 [0.2136,0.2721]	0.0930 [0.0693,0.1167]	0.1622 [0.1231,0.2013]	0.3239 [0.2902,0.3576]	0.0622 [0.0375,0.0868]	0.1159 [0.0907,0.1411]
30000	0.2438 [0.2178,0.2698]	0.0957 [0.0749,0.1166]	0.1621 [0.1279,0.1964]	0.3191 [0.2894,0.3488]	0.0613 [0.0396,0.0830]	0.1179 [0.0956,0.1401]

Table S9. Results of ABC parameters estimation of most probable scenario in 1st model (in grey column median values mentioned in text)

Model 1 - scenario 4								
**Clade C1**								
Parameter	Population trend	mean	median	mode	q025	q050	q950	q975
N4		1.44e+006	1.26e+006	9.62e+005	4.59e+005	5.46e+005	2.93e+006	3.42e+006
t2	↓ **(decline)**	1.50e+004	1.34e+004	9.96e+003	9.20e+003	9.38e+003	2.61e+004	2.82e+004
N4b		6.50e+006	6.86e+006	7.86e+006	3.35e+006	4.01e+006	7.91e+006	7.96e+006
t4	↑ **(growth)**	6.89e+004	6.35e+004	4.89e+004	4.60e+004	4.68e+004	1.08e+005	1.14e+005
N4d		6.49e+004	2.81e+004	2.00e+004	2.00e+004	2.00e+004	2.12e+005	3.31e+005
**Clade C3**								
Parameter	Population trend	mean	median	mode	q025	q050	q950	q975
N4		1.72e+006	1.51e+006	1.15e+006	4.79e+005	5.82e+005	3.69e+006	4.19e+006

t2	↓ **(decline)**	2.02e+004	2.05e+004	2.84e+004	9.65e+003	1.03e+004	2.92e+004	2.96e+004
N4b		7.74e+006	8.09e+006	9.31e+006	3.74e+006	4.42e+006	9.83e+006	9.92e+006
t4	↑ **(growth)**	9.35e+004	9.73e+004	1.14e+005	5.16e+004	5.70e+004	1.18e+005	1.19e+005
N4d		3.32e+004	2.29e+004	1.69e+004	1.09e+004	1.16e+004	7.56e+004	1.07e+005

Table S10 Results of ABC parameters estimation of most probable scenario in 2nd model (in grey column median values mentioned in text)

**Model 2 - scenario 4**								
**Clade 1**								
Parameter	Population trend	mean	median	mode	q025	q050	q950	q975
N4		2.98e+006	2.42e+006	1.12e+006	5.77e+005	7.24e+005	6.96e+006	7.46e+006
t1	↑ **(growth)**	4.22e+003	3.36e+003	1.62e+003	1.09e+003	1.18e+003	1.01e+004	1.10e+004
N4a		1.37e+006	1.11e+006	7.35e+005	2.12e+005	3.05e+005	3.30e+006	3.90e+006
t2	↓ **(decline)**	1.64e+004	1.50e+004	1.08e+004	9.28e+003	9.52e+003	2.72e+004	2.85e+004
N4b		6.43e+006	6.73e+006	7.74e+006	3.35e+006	3.99e+006	7.88e+006	7.94e+006
t4	↑ **(growth)**	8.68e+004	8.76e+004	9.10e+004	5.02e+004	5.38e+004	1.17e+005	1.19e+005
N4d		5.93e+004	4.58e+004	3.39e+004	2.16e+004	2.31e+004	1.38e+005	1.77e+005
**Clade 3**								
Parameter	Population trend	mean	median	mode	q025	q050	q950	q975
N4		2.98e+006	2.44e+006	1.27e+006	5.90e+005	7.42e+005	6.95e+006	7.45e+006
t1	↑ **(growth)**	5.82e+003	5.46e+003	2.57e+003	1.54e+003	1.70e+003	1.11e+004	1.16e+004
N4a		7.89e+005	5.97e+005	3.20e+005	6.55e+004	1.13e+005	2.14e+006	2.72e+006
t2	↓ **(decline)**	1.70e+004	1.59e+004	1.14e+004	9.32e+003	9.63e+003	2.76e+004	2.87e+004
N4b		5.89e+006	6.13e+006	7.94e+006	2.48e+006	3.01e+006	7.84e+006	7.93e+006
t4	↑ **(growth)**	9.95e+004	1.04e+005	1.17e+005	5.69e+004	6.49e+004	1.19e+005	1.19e+005
N4d		1.37e+005	8.05e+004	3.63e+004	1.42e+004	1.83e+004	3.45e+005	4.73e+005

Table S11. Results of ABC parameters estimation of most probable scenario in 3rd model (the grey column shows median values mentioned in text)

**Model 3**								
Parameter	Population trend	mean	median	mode	q025	q050	q950	q975
N1	Na1→N1 ↑ (pop. growth)	8.47e+005	8.15e+005	8.09e+005	3.51e+005	4.14e+005	1.40e+006	1.54e+006
N2	Na2→N2 ↑↑ (pop. growth)	1.35e+006	1.37e+006	1.35e+006	6.63e+005	7.55e+005	1.87e+006	1.93e+006
N3	Na2→N3 ↑↑ (pop. growth)	1.43e+006	1.46e+006	1.49e+006	7.83e+005	8.79e+005	1.89e+006	1.94e+006
t1 (split time)		1.95e+005	1.89e+005	1.89e+005	6.13e+004	7.66e+004	3.38e+005	3.68e+005
Na2	Na1→Na2↑ (pop. growth)	4.44e+005	2.80e+005	4.24e+004	3.16e+004	4.16e+004	1.47e+006	1.70e+006
t2 (split time)		2.32e+005	2.18e+005	1.68e+005	6.97e+004	8.33e+004	4.32e+005	4.65e+005
Na1	ancestral population	3.69e+005	2.35e+005	5.72e+004	2.96e+004	3.94e+004	1.20e+006	1.47e+006

↑ - small/moderate population growth; ↑↑ - high population growth

Figure S2. Demographic and evolutionary scenarios within the three models using the ABC method implemented in DIYABC.

Model 1

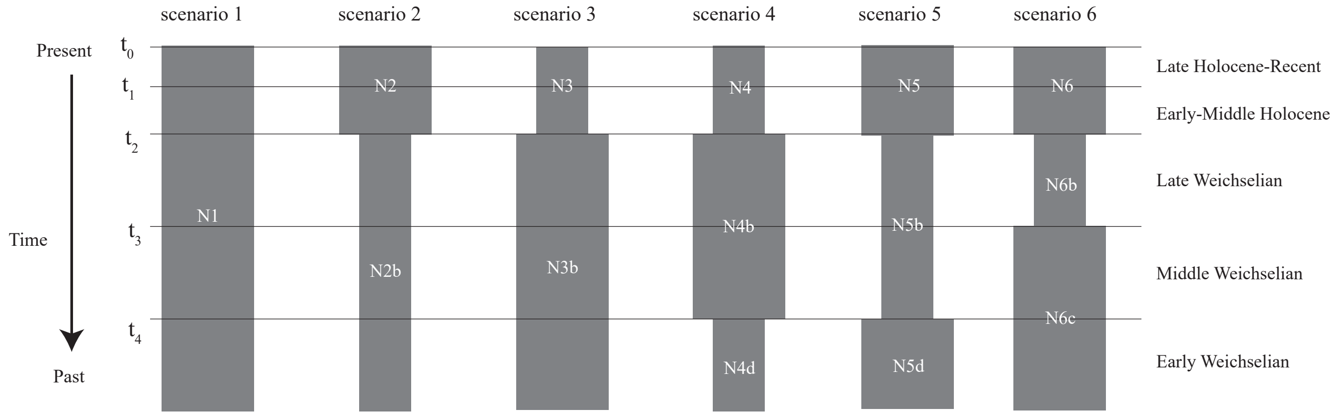

Model 2

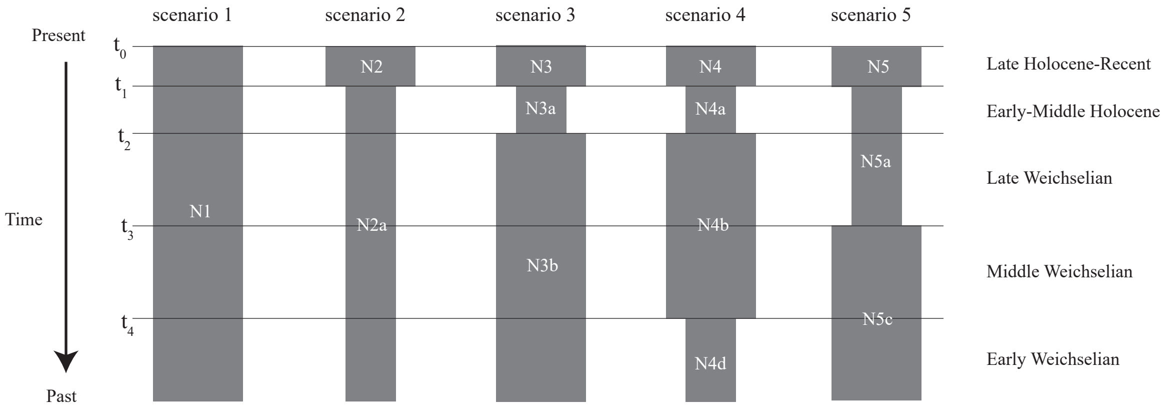

Model 3

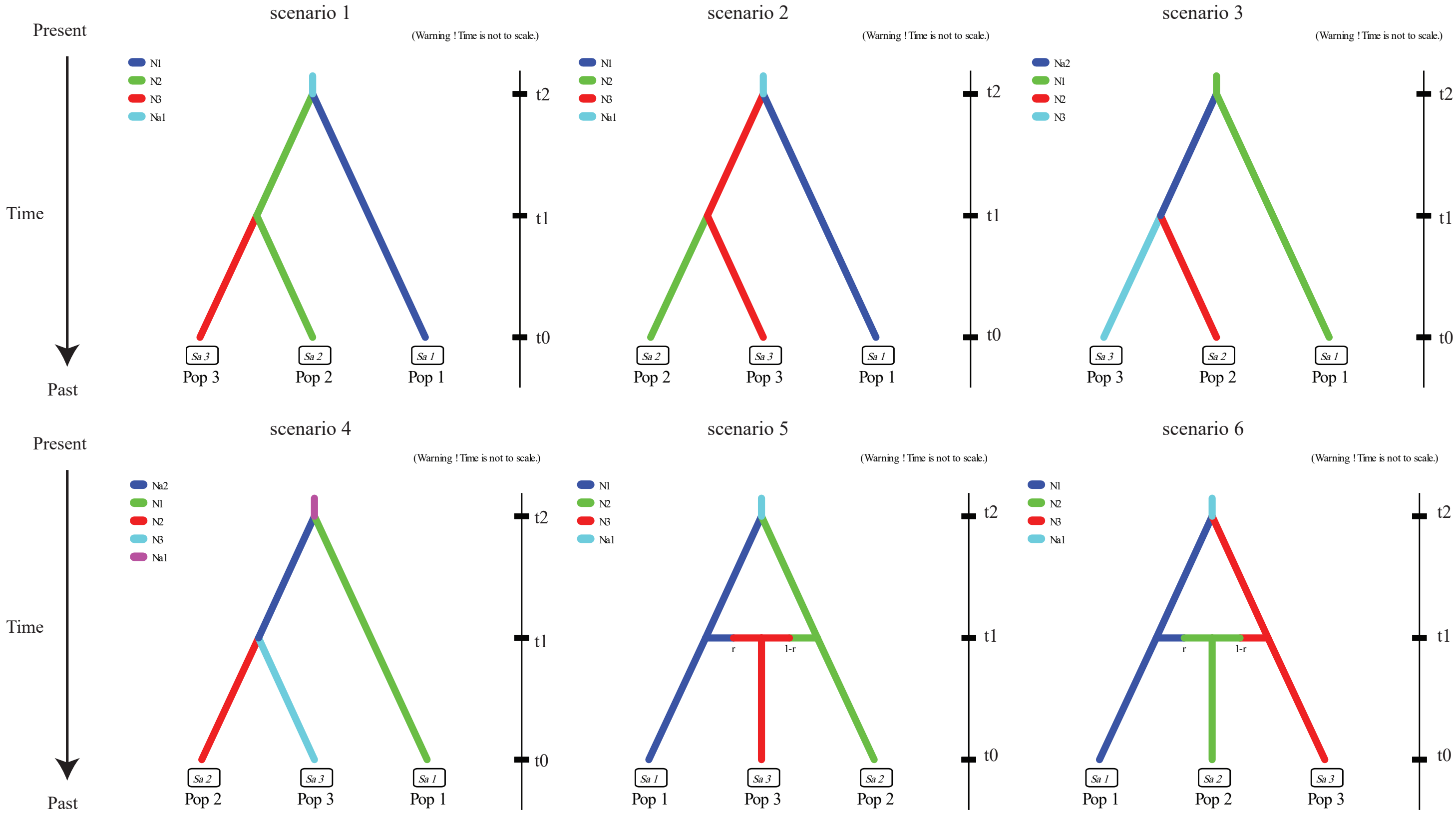

N1/Pop 1 - clade C5 - mainland; N2/Pop 2: clade C3 and C5 South Korea - remote islands; N3/Pop 3: clade C3 - South Korea - peninsula
Na - ancestral populations
