# Supplementary material for: Late Pleistocene Expansion of Small Murid Rodents across the Palearctic in Relation to the Past Environmental Changes
Source: Genes (Basel). 2021 Apr 26;12(5):642. doi: 10.3390/genes12050642 (PMC8145813; doi:10.3390/genes12050642)
Supplement: Supplementary file 1 [file genes-12-00642-s001.zip › File S4.pdf]

Late Pleistocene Expansion of Small Murid Rodents across the Palearctic in Relation to the Past Environmental Changes

Kozyra K, Zając T.M., Ansorge H., Wierzbicki H., Moska M., Stanko M., Stopka P.

Supplementary materials S4
Modeling species distribution

To estimate the historical distribution range of the SFM during the Last Glacial Maximum, we employed environmental niche modelling (ENM), using the maximum entropy method implemented in MaxEnt software ver. 3.4 [1]. MaxEnt reconstructs the distribution of a species based on presence data used as georeferenced point occurrences, and on annexed environmental condition data used as variables determining the species' occurrence. We obtained current verified SFM records from the Eurasia, which we used to fit a species distribution model (SDM) from various sources: the GBIF database; published data [2–8]; and unpublished data via personal communication. Environmental data regarding actual and historical bioclimatic conditions were retrieved from the World Bioclim database, ver. 1.4. (http://www.worldclim.org), with a resolution of 2.5'[9]. Topographic layers during the Last Glacial Maximum (LGM) have been reconstructed on the basis of SRTM30_PLUS Global Bathymetry layers [10,11], available from published data on sea level changes during the Pleistocene [12,13].

To avoid problems with collinearity among 21 bioclimatic variables, we eliminated highly correlated variables. To detect these, we estimated Pearson's correlation coefficients using the SDMtoolbox in ArcGIS (Table S12). When a pair of variables was as strongly correlated, with the correlation coefficients at least 0.9, the tool removes one of the paired variables. The final set of variables to be used in modelling niches, obtained after this procedure, included twelve bioclimatic and three topographic variables (Table S13).

To reduce occurrence data to single points within the specified 50 km distance, we prepared the georeferenced records of the species using the "spatially rarefy occurrence data" tool of the SDMtoolbox tool in ArcGIS 10.1. Spatially independent localities should be used to improve the model performance, and to avoid model overfitting due to environmental bias [14–16]. In order to do so, MaxEnt randomly partitions the species' occurrences and, using the cross-validation resampling method, evaluates the accuracy of the model's fit to those partitions. A model's accuracy is presented as the Area Under Curve (AUC) value of the Receiver Operating Characteristic (ROC), giving an estimated value, representing the model's accuracy, ranging from 0 to 1 [17] (Table S14). An AUC of 0.5 represents random probability, which means that the species' occurrences are random. Values below 0.5 suggests that the model performs worse than a model with random partition. AUC values above 0.5 indicates the model is well-fitted, and values close or equal to 1 indicate a perfect fit, which means the model perfectly represents the spatial distribution of the species. Models with an AUC above 0.75 are considered good models, and those with an AUC over 0.90 excellent [18,19]. The estimated areas of the species' distribution during the LGM can be interpreted, especially in the temperate climate zone, as the refugia that members of the species would most likely use under the most unfavorable climatic conditions. All of the analyses were performed to

reconstruct the historical distributions and distinct populations (potentially capable of showing distinct habitat/climate preferences) of the SFMs along the whole Eurasia, with the confirmed strong signal of a recent expansion during the Last (Weichselian) glaciation modeled separately.

References

1. Phillips, S.J.; Dudík, M. Modeling of species distributions with Maxent: new extensions and a comprehensive evaluation. *Ecography (Cop.).* **2008**, *31*, 161–175, doi:10.1111/j.0906-7590.2008.5203.x.

2. Herzig-Straschil, B.; Bihari, Z.; Spitzenberger, F. Recent changes in the distribution of the field mouse (Apodemus agrarius) in the western part of the Carpathian basin. *Ann. des Naturhistorischen Museums Wien* **2004**, *105B*, 421–428.

3. Kartavtseva, I. V.; Pavlenko, M. V. Chromosome variation in the striped field mouse Apodemus agrarius (Rodentia, Muridae). *Russ. J. Genet.* **2000**, *36*, 162–174.

4. Musser, G.G.; Brothers, E.M.; Carleton, M.D.; Hutterer, R. Taxonomy and distributional records of Oriental and European Apodemus, with a review of the Apodemus-Sylvaemus problem. *Bonner Zool. Beitraege* **1996**, *46*, 143–190.

5. Sakka, H.; Quéré, J.-P.; Kartavtseva, I.; Pavlenko, M.; Chelomina, G.; Atopkin, D.; Bogdanov, A.; Michaux, J. Comparative phylogeography of four Apodemus species (Mammalia: Rodentia) in the Asian Far East: evidence of Quaternary climatic changes in their genetic structure. *Biol. J. Linn. Soc.* **2010**, *100*, 797–821, doi:10.1111/j.1095-8312.2010.01477.x.

6. Stanko, M. *Apodemus agrarius (Pallas 1771) (Rodentia, Muridae) in Slovakia*; Equilibria s.r.o. (in Slovakian with English summary).: Kosice, 2014;

7. Tulis, F.; Ambros, M.; Balaž, I.; Žiak, D.; Hulejová Sládkovičová V.; Miklós, P.; Dudich, A.; Stollmann, A. Expansion of the Striped field mouse (Apodemus agrarius) in the south-western Slovakia during 2010–2015. *Folia Oecologica* **2016**, *43*, 67–73.

8. Weber, B. Zur Verbreitung und Ökologie der Brandmaus, Apodemus agrarius (Pallas, 1771), im westlichen Mecklenburg, in der Altmark und der Magdeburger Börde. *Hercynia* **1972**, 302–308.

9. Hijmans, R.J.; Cameron, S.E.; Parra, J.L.; Jones, P.G.; Jarvis, A. Very high resolution interpolated climate surfaces for global land areas. *Int. J. Climatol.* **2005**, *25*, 1965–1978, doi:10.1002/joc.1276.

10. Smith, W.H.F.; Sandwell, D.T. Global Sea Floor Topography from Satellite Altimetry and Ship Depth Soundings. *Science (80-.).* **1997**, *277*, 1956–1962, doi:10.1126/science.277.5334.1956.

11. Becker, J.J.; Sandwell, D.T.; Smith, W.H.F.; Braud, J.; Binder, B.; Depner, J.; Fabre, D.; Factor, J.; Ingalls, S.; Kim, S.H.; et al. Global Bathymetry and Elevation Data at 30 Arc Seconds Resolution: SRTM30_PLUS. *Mar. Geod.* **2009**, *32*, 355–371, doi:10.1080/01490410903297766.

12. Bintanja, R.; van de Wal, R.S.W.; Oerlemans, J. Modelled atmospheric temperatures and global sea levels over the past million years. *Nature* **2005**, *437*, 125–8, doi:10.1038/nature03975.

13. Bintanja, R.; van de Wal, R.S.W. North American ice-sheet dynamics and the onset of 100,000-year glacial cycles. *Nature* **2008**, *454*, 869–72, doi:10.1038/nature07158.

14. Boria, R.A.; Olson, L.E.; Goodman, S.M.; Anderson, R.P. Spatial filtering to reduce sampling bias can improve the performance of ecological niche models. *Ecol. Modell.* **2014**, *275*, 73–77, doi:10.1016/j.ecolmodel.2013.12.012.

15. Hijmans, R.J. Cross-validation of species distribution models: removing spatial sorting bias and calibration with a null model. *Ecology* **2012**, *93*, 679–688, doi:10.1890/11-0826.1.

16. Veloz, S.D. Spatially autocorrelated sampling falsely inflates measures of accuracy for presence-only niche models. *J. Biogeogr.* **2009**, *36*, 2290–2299, doi:10.1111/j.1365-2699.2009.02174.x.

17. Phillips, S.J.; Anderson, R.P.; Schapire, R.E. Maximum entropy modeling of species geographic distributions. *Ecol. Modell.* **2006**, *190*, 231–259, doi:10.1016/j.ecolmodel.2005.03.026.

18. Swets, J.A. Measuring the accuracy of diagnostic systems. *Science* **1988**, *240*, 1285–93.

19. Elith, J.; H. Graham, C.; P. Anderson, R.; Dudík, M.; Ferrier, S.; Guisan, A.; J. Hijmans, R.; Huettmann, F.; R. Leathwick, J.; Lehmann, A.; et al. Novel methods improve prediction of species' distributions from occurrence data. *Ecography (Cop.).* **2006**, *29*, 129–151, doi:10.1111/j.2006.0906-7590.04596.x.

Table S12. Correlation coefficient between the climatic variables used in Maxent analysis.

	BIO1	BIO2	BIO3	BIO4	BIO5	BIO6	BIO7	BIO8	BIO9	BIO10	BIO11	BIO12	BIO13	BIO14	BIO15	BIO16	BIO17	BIO18	BIO19
BIO1																			
BIO2	0,42																		
BIO3	0,82	0,39																	
BIO4	-0,86	-0,14	-0,86																
BIO5	0,89	0,62	0,61	-0,54															
BIO6	**0,97**	0,25	0,85	**-0,95**	0,77														
BIO7	-0,78	0,06	-0,79	**0,98**	-0,41	**-0,9**													
BIO8	0,74	0,38	0,59	-0,51	0,74	0,65	-0,43												
BIO9	**0,94**	0,36	0,77	-0,85	0,81	**0,94**	-0,79	0,52											
BIO10	**0,93**	0,53	0,65	-0,62	**0,99**	0,83	-0,52	0,77	0,85										
BIO11	**0,98**	0,33	0,86	**-0,94**	0,79	**1**	-0,88	0,67	**0,94**	0,85									
BIO12	0,26	-0,39	0,41	-0,43	0	0,36	-0,51	0,21	0,21	0,09	0,33								
BIO13	0,33	-0,19	0,42	-0,43	0,12	0,38	-0,46	0,33	0,25	0,2	0,37	0,89							
BIO14	-0,04	-0,53	0,11	-0,16	-0,24	0,08	-0,28	-0,08	-0,02	-0,17	0,03	0,61	0,27						
BIO15	0,41	0,64	0,39	-0,26	0,47	0,31	-0,12	0,5	0,3	0,44	0,37	-0,12	0,18	-0,51					
BIO16	0,31	-0,22	0,4	-0,43	0,1	0,37	-0,46	0,3	0,23	0,17	0,36	**0,92**	**0,99**	0,31	0,13				
BIO17	-0,01	-0,54	0,15	-0,19	-0,22	0,12	-0,32	-0,06	0,01	-0,15	0,06	0,66	0,32	**0,99**	-0,5	0,36			
BIO18	0	-0,38	0,1	-0,13	-0,19	0,06	-0,21	0,15	-0,1	-0,11	0,04	0,79	0,71	0,48	-0,09	0,74	0,51		
BIO19	0,22	-0,28	0,41	-0,38	0,02	0,32	-0,44	0,05	0,25	0,08	0,29	0,66	0,49	0,56	-0,22	0,51	0,59	0,28	

Table S13. Variables used in reconstruction an past environmental niche models of *A. agrarius* in Maxent and percent contribution of variables used in model building.

Code	Variable	Percent contribution				
		C1	C2	C3	C4	C5
**BIO1**	Annual Mean Temperature	**47.4**	**16.4**	**6.9**	1.7	**17.9**
**BIO2**	Mean Diurnal Range (Mean of monthly (max temp - min temp))	0.2	0.1	0.2	0.3	0.1
**BIO3**	Isothermality (BIO2/BIO7) (* 100)	1.3	1	0.2	1.2	1.3
**BIO4**	Temperature Seasonality (standard deviation *100)	4.3	**22.1**	**18.5**	**19.3**	**14.8**
**BIO5**	Max Temperature of Warmest Month	1.2	0.2	0.9	**12.2**	2.3
**BIO8**	Mean Temperature of Wettest Quarter	**13.6**	0.7	2.8	1.7	1.2
**BIO12**	Annual Precipitation	3	0.1	0.3	0.1	0
**BIO13**	Precipitation of Wettest Month	0.3	3.5	**15.3**	**10.8**	**7.1**
**BIO14**	Precipitation of Driest Month	**14.4**	0.4	0.3	0.3	0.1
**BIO15**	Precipitation Seasonality (Coefficient of Variation)	1.2	**10.9**	5.3	0.6	3.2
**BIO18**	Precipitation of Warmest Quarter	**7.4**	**37.4**	**41.8**	**49.2**	**36.8**
**BIO19**	Precipitation of Coldest Quarter	0.2	4.3	0	0.4	**13.4**
**alt**	Altitude	4.9	1.9	**6.9**	1.8	0.2
**tpi**	Topographic Position Index	0.3	0	0.6	0	0.1
**topo_het**	Topographic Heterogenity	0.3	0.9	0.3	0.4	1.4

Table S14. Measurement the accuracy of Maxent models of reconstructed predicted present and past distribution range of *A. agrarius* clades.

AUC statistic – results	C1	C2	C3	C4	C5
Training AUC	0.9306	0.9874	0.9912	0.9850	0.9897
Test AUC	0.9239	0.9813	0.9880	0.9820	0.9863
AUC Standard Deviation	0.0149	0.0073	0.0054	0.0060	0.0049

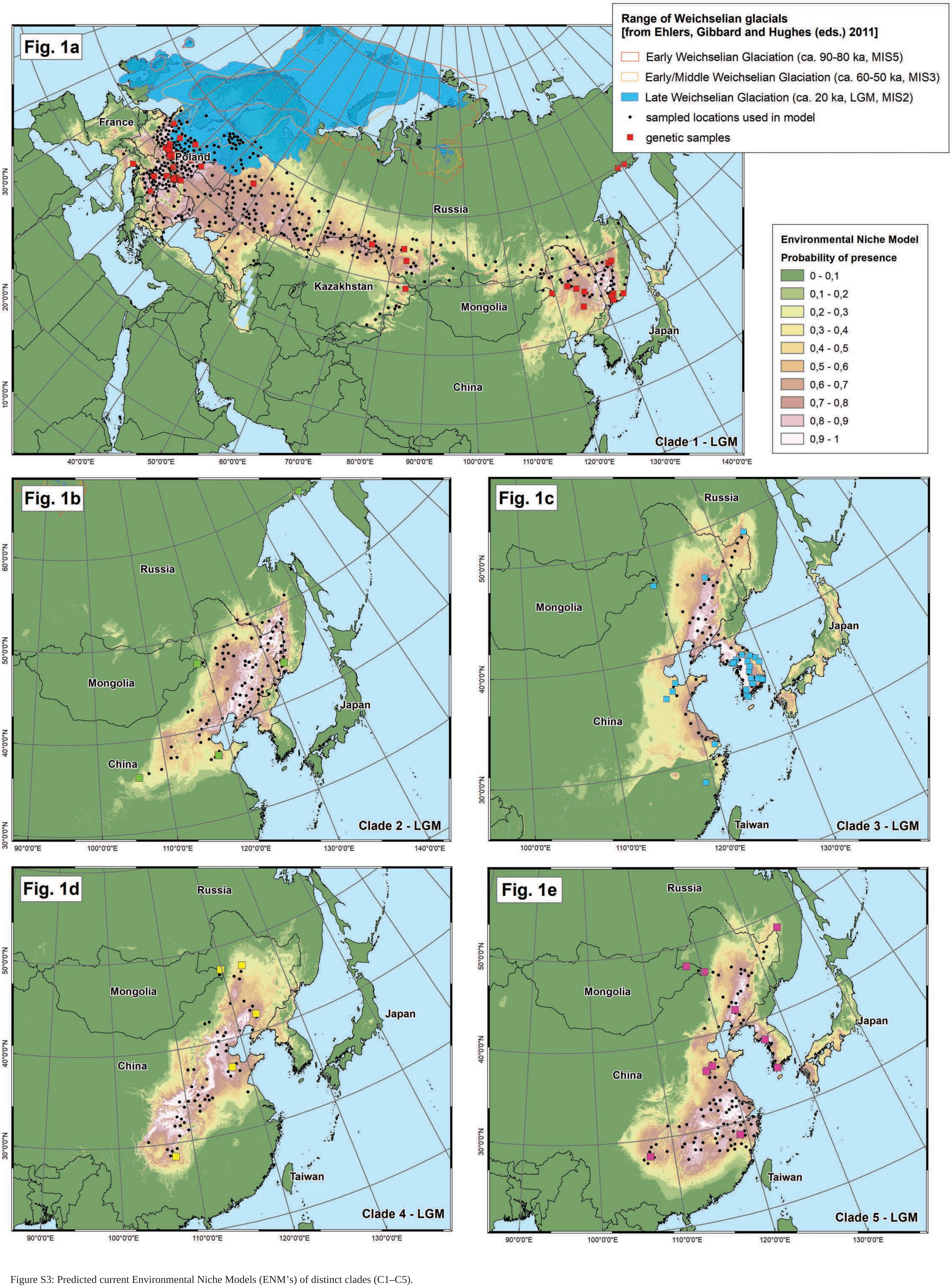

Figure S3: Predicted current Environmental Niche Models (ENM's) of distinct clades (C1–C5).

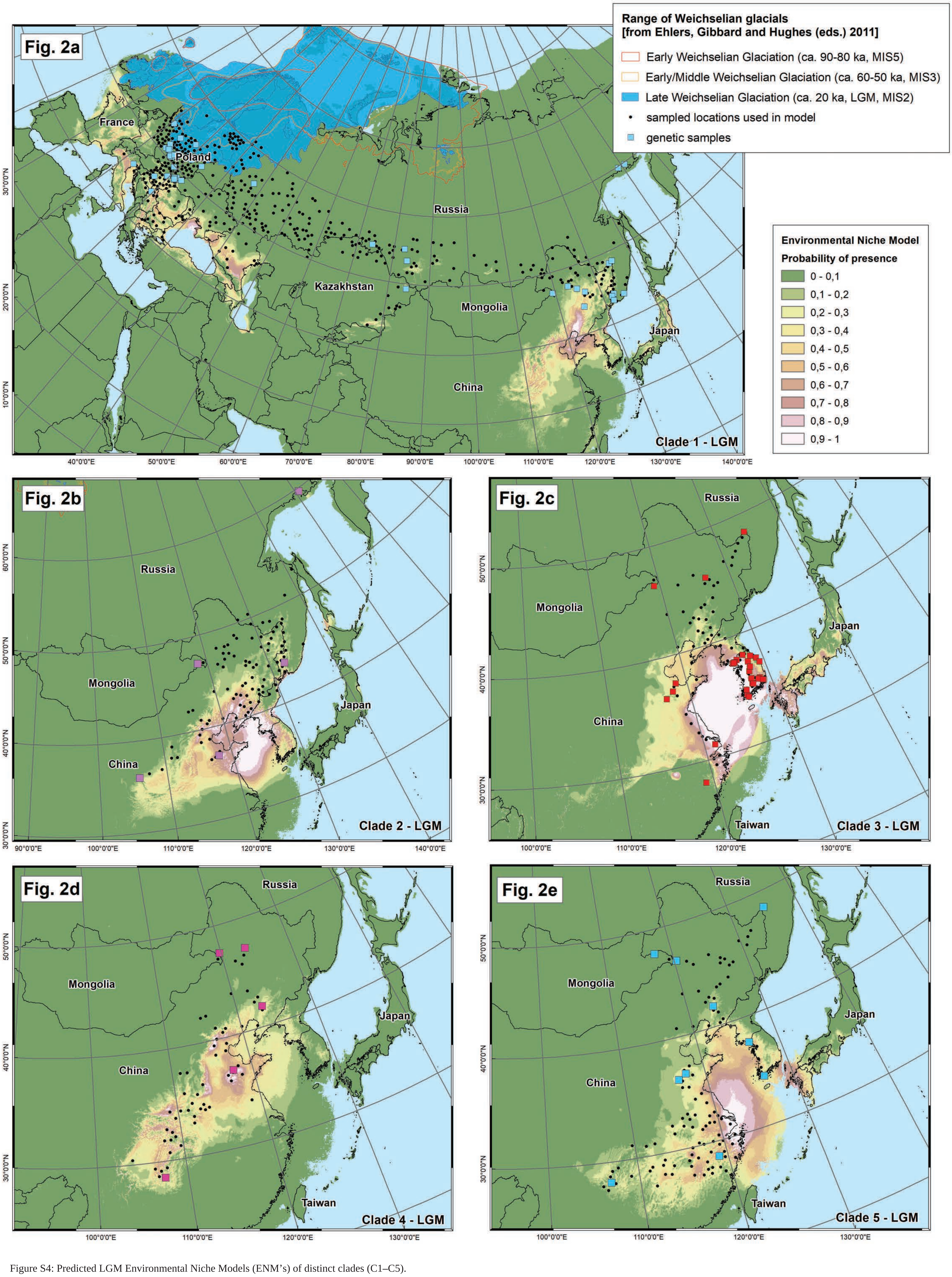

Figure S4: Predicted LGM Environmental Niche Models (ENM's) of distinct clades (C1–C5).
