# Supplementary material for: Late Pleistocene Expansion of Small Murid Rodents across the Palearctic in Relation to the Past Environmental Changes
Source: Genes (Basel). 2021 Apr 26;12(5):642. doi: 10.3390/genes12050642 (PMC8145813; doi:10.3390/genes12050642)
Supplement: Supplementary file 1 [file genes-12-00642-s001.zip › File S5.pdf]

Late Pleistocene Expansion of Small Murid Rodents across the Palearctic in Relation to the Past Environmental Changes

Kozyra K., Zając T. M., Ansorge H., Wierzbicki H., Moska M., Stanko M., Stopka P.

Supplementary Materials S5

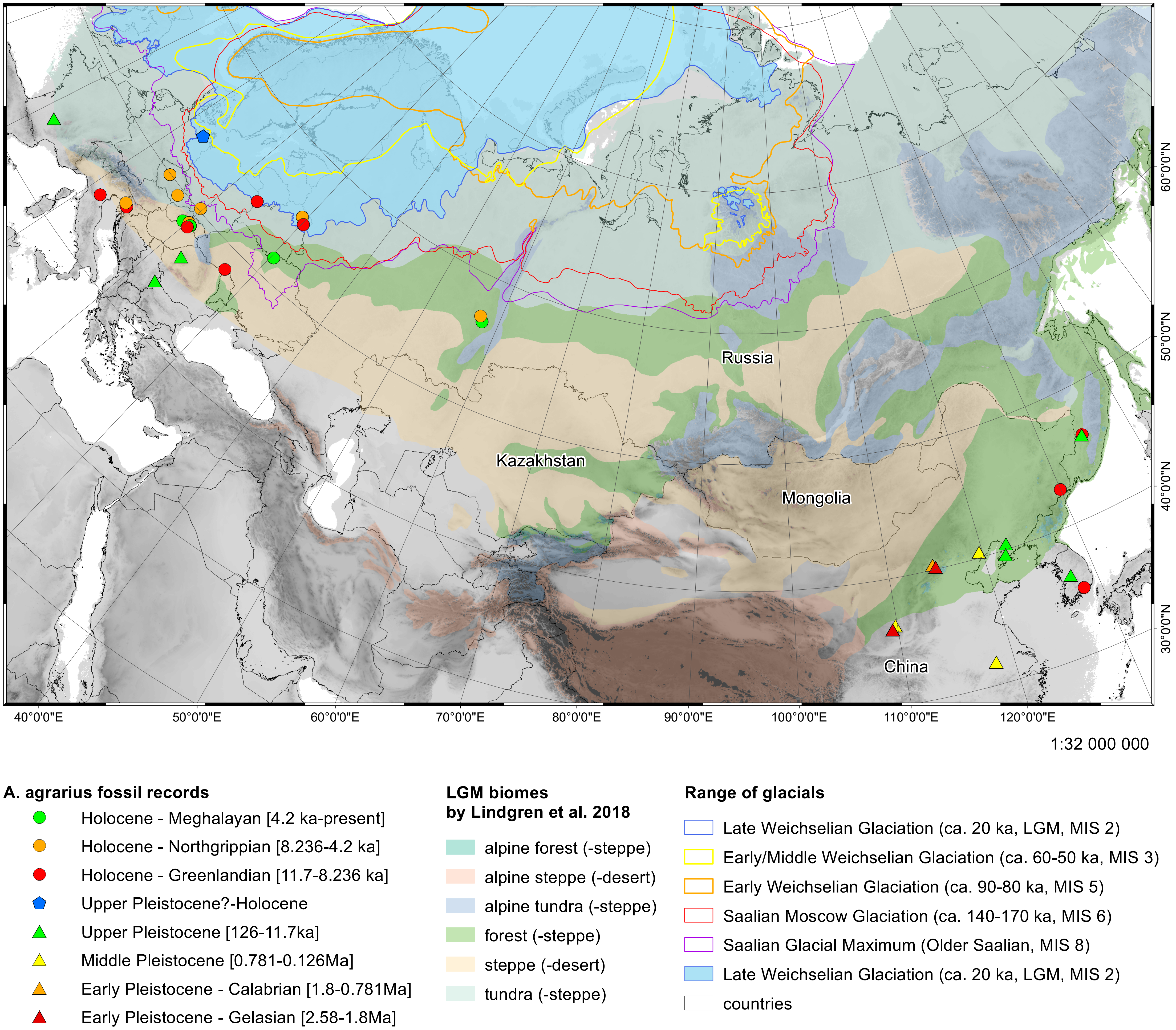

Figure S5 The range of glacials during the Weichselian and Saalian glaciation and range of biomes during the Last Glacial Maximum, with fossil records of striped-field mouse *Apodemus agrarius* in the Palearctic were based on published data Aguilar et al., 2008; Ivanov, 2016; Izvarin & Ulitko, 2018; Jin & Kawamura, 1996; Kawamura, 1989; Knitlová & Horáček, 2017; Kotsakis et al., 2003; Kowalski, 2001; Popov, 2017; Zhang, Li, Wang, & Gong, 2010.
