# Supplementary material for: Late Pleistocene Expansion of Small Murid Rodents across the Palearctic in Relation to the Past Environmental Changes
Source: Genes (Basel). 2021 Apr 26;12(5):642. doi: 10.3390/genes12050642 (PMC8145813; doi:10.3390/genes12050642)
Supplement: Supplementary file 1 [file genes-12-00642-s001.zip › File S6.pdf]

Late Pleistocene Expansion of Small Murid Rodents across the Palearctic in Relation to the Past Environmental Changes

Kozyra K., Zając T. M., Ansorge H., Wierzbicki H., Moska M., Stanko M., Stopka P.

Supplementary Materials S6

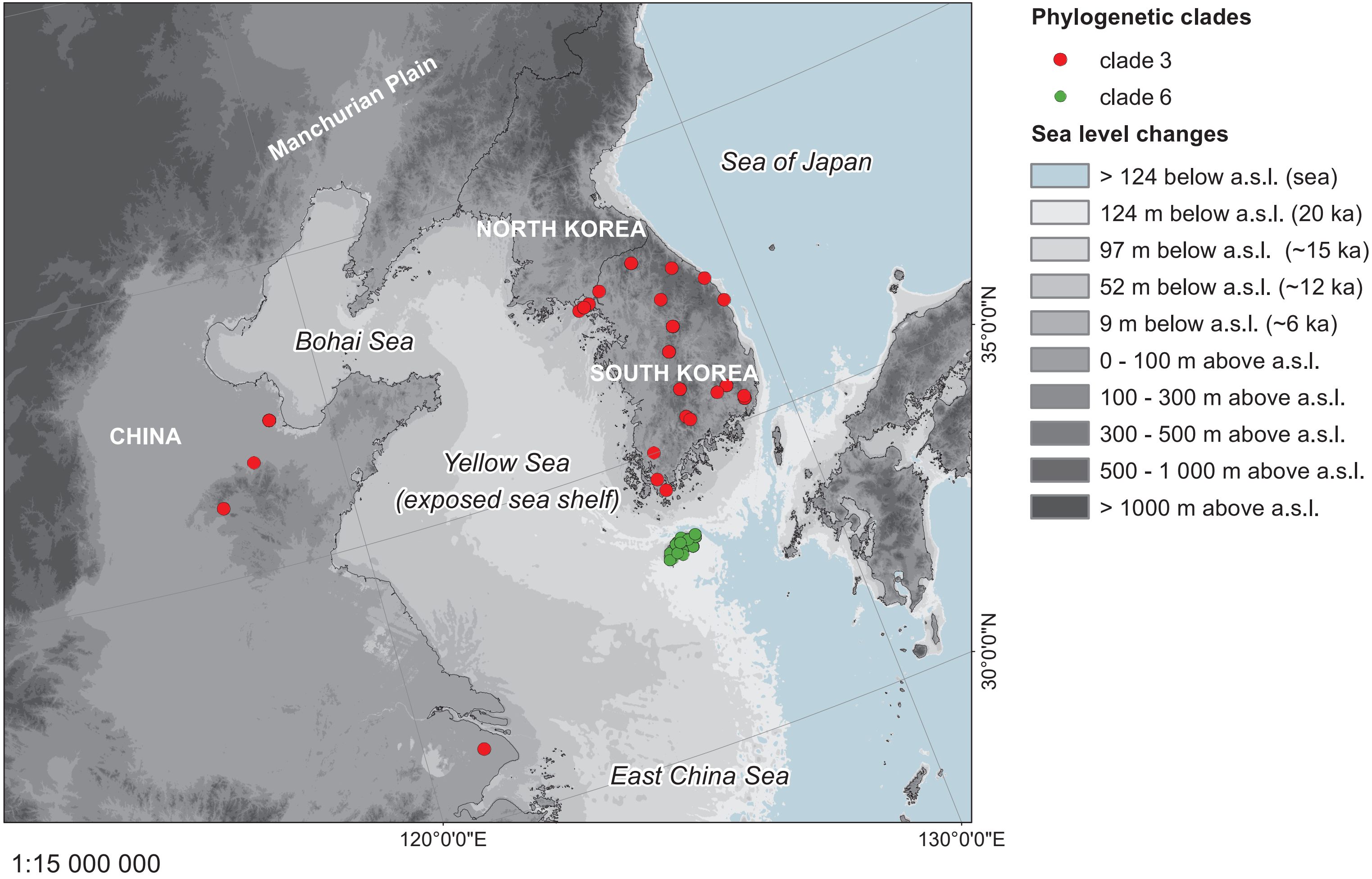

Figure S6. Reconstructed sea level changes during the LGM and post-glacial period in southeast Asia and formation of exposed Bohai and Yellow Sea Shelf.
