# Supplementary material for: Late Pleistocene Expansion of Small Murid Rodents across the Palearctic in Relation to the Past Environmental Changes
Source: Genes (Basel). 2021 Apr 26;12(5):642. doi: 10.3390/genes12050642 (PMC8145813; doi:10.3390/genes12050642)
Supplement: Supplementary file 1 [file genes-12-00642-s001.zip › File S7.pdf]

Kozyra K., Zając T. M., Ansorge H., Wierzbicki H., Moska M., Stanko M., Stopka P.

Supplementary Materials S7

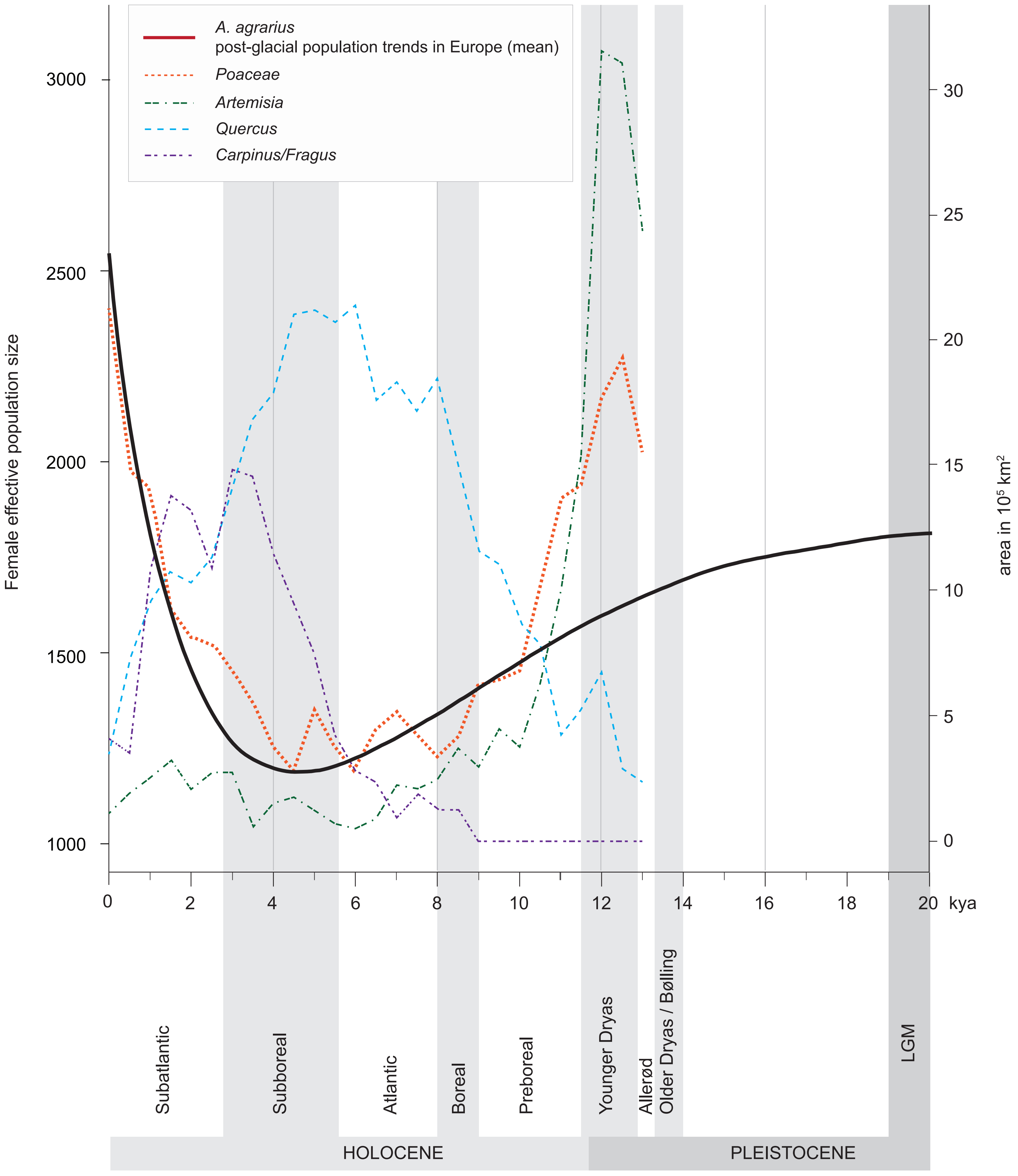

Figure S7. Post-glacial demography trends of striped-field mouse in relation to changes in area of highest abundance for the most common European trees and plant communbities reconstructed by Giesecke et al. (2017).
